# Extracellular DNA in blood products and its potential effects on transfusion

**DOI:** 10.1042/BSR20192770

**Published:** 2020-03-25

**Authors:** Li Yang, Dongmei Yang, Qian Yang, Fu Cheng, Yuanshuai Huang

**Affiliations:** 1 Department of Transfusion, The Affiliated Hospital of Southwest Medical University, 25 Tai Ping Road, Jiang Yang District, Luzhou 646000, China; 2 Department of Transfusion, The West China Hospital, Sichuan University, 37 Guoxue Lane, Wuhou District, Chengdu 610041, China

**Keywords:** extracellular DNA, horizontal gene transfer, innate immune response, mtDNA, transfusion adverse reaction, transfusion

## Abstract

Blood transfusions are sometimes necessary after a high loss of blood due to injury or surgery. Some people need regular transfusions due to medical conditions such as haemophilia or cancer. Studies have suggested that extracellular DNA including mitochondrial DNA present in the extracellular milieu of transfused blood products has biological actions that are capable of activating the innate immune systems and potentially contribute to some adverse reactions in transfusion. From the present work, it becomes increasingly clear that extracellular DNA encompassed mitochondrial DNA is far from being biologically inert in blood products. It has been demonstrated to be present in eligible blood products and thus can be transfused to blood recipients. Although the presence of extracellular DNA in human plasma was initially detected in 1948, some aspects have not been fully elucidated. In this review, we summarize the potential origins, clearance mechanisms, relevant structures, and potential role of extracellular DNA in the innate immune responses and its relationship with individual adverse reactions in transfusion.

## Introduction

The presence of extracellular DNA (ecDNA) was first reported by Mandel and Métais [1] in 1948. The term ecDNA describes any DNA existing in the extracellular environment, regardless of structure (association with protein complexes and extracellular vesicles) [2,3]. Although another term, cell-free DNA (cfDNA), is widely used presently, the term ecDNA is more accurate in this review because a large proportion of DNA *in vivo* is localized in complexes or is packaged in vesicles rather than being truly free [4,5]. Found in the extracellular milieu including serum, plasma, lymph, bile, milk, urine, saliva, spinal fluid, amniotic fluid, and cerebrospinal fluid, ecDNA can be isolated from individuals in both healthy and various disease states [6–10]. ecDNA comprises mainly nuclear DNA (nucDNA) and mitchondrial DNA (mitDNA). It is also present in the extracellular milieu of blood products (as shown in Table 1) [11–20]. However, a novel and significant role of ecDNA has emerged, involving its ability to trigger innate immune system responses and drive inflammation when released from mechanically injured cells [21]. ecDNA, along with other host molecules released upon cell damage, falls into the category of damage-associated molecular patterns (DAMPs) [22,23].

**Table 1:** Extracellular DNA in blood products and their concentration

Reference	Blood products	DNA concentration	Sample number	DNA type	Quantitative method
Duxbury et al., 1995 [11]	Whole blood	Range 260–1474 ng/ml	7	total DNA	Threshold total DNA Assay system
Dijkstra-Tiekstra et al., 2004 [12]	PCs (before filtration)	1.7 ± 0.8 leucocyte-eq/μl	5	nucDNA	Quantitative real-time polymerase chain reaction
	PCs (after fitration)	1.5± 0.8 leucocyte-eq/μl	5		
Ivancic-Jelecki et al., 2009 [13]	Plasma	Range 0.06–22.5 ng/ml	10	nucDNA	Quantitative real-time polymerase chain reaction
Lee et al., 2014 [14]	RBCUs (LR)	Range 0.8–87-fold	11	mitDNA	Quantitative real-time polymerase chain reaction
	PCs	Range 0.4–235.6-fold	5		
	FFP	Range 0.7–46.5-fold	16		
Cognasse et al., 2016 [15]	AE-associated PCs	484 ± 313.45 ng/ml (peak concentration)	42	mitDNA	Quantitative real-time polymerase chain reaction
	Control PCs	122.55 ± 52.64 ng/ml (peak concentration)	59		
Shih et al., 2016 [16]	RBCUs (WBF)	1.08 ± 0.90 ng/ml	44	total DNA	PicoGreen assay
	RBCUs (WBF)	3.57 ± 1.99 μg/ml	47		Spectrophotometry
	RBCUs (RCF)	0.50 ± 0.77 ng/ml	73		PicoGreen assay
	RBCUs (RCF)	3.28 ± 1.28 μg/ml	73		Spectrophotometry
Bakkour et al., 2016 [17]	RBCUs (non-LR)	5.3 × 10^5^ copies/μl (mean concentration)	12	mitDNA	Quantitative real-time polymerase chain reaction
	RBCUs (MCS+ apheresis)	1.3 × 10^5^ copies/μl (mean concentration)	12		
	RBCUs (Trima apheresis)	1.2 × 10^5^ copies/μl (mean concentration)	12		
Yashui et al., 2016 [18]	NHTR-associated PCs	Range 1.4–110 × 10^4^ copies/ml	17	mitDNA	Quantitative real-time polymerase chain reaction
	NHTR-associated RBCUs	Range 0.3–23 × 10^4^ copies/ml	20		
	NHTR-associated FFP	Range 2.3–18 × 10^4^ copies/ml	11		
	Control PCs	Range 0.1–3.8 × 10^4^ copies/ml	320		
Simmons et al., 2017 [19]	RBCUs	3 ± 0.4 ng/ml	114	mitDNA	Quantitative polymerase chain reaction
	PCs	94.8 ± 69.2 ng/ml	17		
	FFP	213.7 ± 65 ng/ml	81		
Waldvogel Abramowski et al., 2018 [20]	RBCUs	290 ± 120 ng/ml	23	total DNA	Qubit Fluorometer
	PCs	339.6 ± 114 ng/ml	23		
	FFP	2.875 ± 0.996 ng/ml	23		

## ecDNA in blood products

Blood can be transfused without prior modification (whole-blood transfusion) or divided into red blood cell units (RBCUs), fresh frozen plasma (FFP), platelet concentrates (PCs) and sometimes granulocytes. These blood products for therapeutic use usually contain donors’ plasma, platelets, residual leukocytes and erythrocytes. The DNA of a donor can, therefore, be transferred to a recipient via ecDNA in the plasma fluid, ecDNA bound to the surfaces of blood components (such as erythrocytes and platelets) or via the DNA localized in complexes or packaged in vesicles. According to the report of García-Olmo et al. [24], ecDNA from human plasma can pass through the 0.4 micron filters of Corning Transwell plates and there were no significant differences between the effects of human plasma administered to cell cultures indirectly through the Transwell plates and plasma administered directly to the cells, indicating that no DNA was lost due to the filter.

As early as in 1995, detectable DNA has been shown to be present in stored human donor blood at levels in the range of 250–1500 ng/ml with a Threhold Total DNA Assay Kit, and the total amount of DNA administered to a patient during the transfusion of a single unit of whole blood can be as much as 450 μg (based on 1.5 μg DNA/ml of CPD plasma and assuming a plasma volume of 60% in a 500 ml unit of blood) [11]. According to studies in the subsequent years, ecDNA was also observed in the plasma of transfusion blood components including RBCUs, PCs, and FFP [12–20]. The studies on ecDNA in transfusion products and their concentration are summarized in Table 1.

As can be seen from the table, in the report of Waldvogel Abramowski et al. [20], cellular products including RBCUs (290  ±  120 ng/ml) and PCs (339.6 ± 114 ng/ml) contained more ecDNA than did FFP (2.875 ± 0.996 ng/ml), suggesting a potential link with the number of cells found in the blood bag. Simmons et al. [19] found detectable levels of extracellular mitDNA in FFP (213.7 ± 65 ng/ml), PCs (94.8 ± 69.2 ng/ml), and RBCUs (3 ± 0.4 ng/ml). In the present study, the concentration of extracellular mitDNA detected in RBCUs was lowest among the three tested, perhaps because RBCs do not contain mitochondria and are subjected to leukocyte reduction [19]. Of note, the generation or release of ecDNA present in blood products could be influenced by the details of the processing and manufacturing methods of blood components [16,17]. Shih et al. [16] have shown that that the levels of ecDNA are affected by RBCUs processing method as well as product age: whole blood filtered (WBF), short-term storage RBCUs had more ecDNA than red cell filtered (RCF), aged RBCUs. However, according to the study of Dijkstra-Tiekstra et al. [12], the amount of ecDNA was not influenced by filtration of the PCs (1.7 ± 0.8 vs. 1.5 ± 0.8 leucocyte-eq/μl).

Unfortunately, many studies with respect to ecDNA in blood products were performed with dissimilar analytical procedures, rendering comparison with their results infeasible. Some of these research results do not differentiate between nucDNA and mitDNA that are thought to be of separate evolutionary origins [25,26]. MitDNA consists of a high number of unmethylated CpG islands, as typically found in bacteria [27].

## Potential origins and clearance of ecDNA in blood products

### Potential origins

To date, no consensus has yet been reached with regard to the main origin of ecDNA. However, there are two contenders for the main origin of ecDNA: (i) cellular breakdown mechanisms and (ii) active release mechanisms [28]. Cellular breakdown mechanisms such as apoptosis and necrosis are considered to be the main processes for producing ecDNA by some researchers [29,30]. It may be of interest to note that other forms of cell death such as NETosis can also serve as sources for ecDNA [31]. Numerous studies have demonstrated that ecDNA can also be derived from active release mechanisms [32–35]. However, it is still unclear which mechanism accounts for the ecDNA observed in blood products.

During storage, cellular breakdown mechanisms, such as necrosis, apoptosis and NETosis, could theoretically lead to ecDNA release in blood products. Neutrophils can release both nucDNA and mitDNA in structures known as neutrophil extracellular traps (NETs), which are composed of decondensed chromatin decorated with granular proteins [36–38]. The formation of NETs requires the activation of neutrophils and the release of their DNA in a process that may or may not result in neutrophil death. Additionally, ecDNA could be encapsulated in extracellular vesicles (EVs), such as microparticles (MPs) [39], which have also been detected in blood products [40,41]. This ecDNA, present in membrane-bound EVs, can be protected from nuclease mediated degradation and can be released through the breakdown of EVs [4].

What’s more, activated platelets have been reported to release intact mitochondria, which can be hydrolysed by group IIA secretory phospholipase A2 (sPLA2-IIA), and thereby release extracellular mitDNA [42]. In their study, Boudreau et al. [42] demonstrated that inactivated platelets contain an average of ∼4 mitochondria using fluorescence and transmission electron microscopy.

### Clearance mechanisms

Since 1966, work on autoimmune pathologies has permitted the first characterization of ecDNA [43–46], it is resistant to RNase and proteinase K [47], but can be hydrolyzed by DNase. Studies on ecDNA clearance revealed that extracellular DNases such as DNase1 and DNase1L3 could degrade ecDNA associated with a variety of structures [48–52]. DNase1 and DNase1l3, which have close structural and functional resemblance, may substitute or cooperate with each other during DNA degradation [50]. The enzyme DNase1 plays a role in the clearance of chromatin during necrosis [49]. DNase1L3 is uniquely capable of digesting chromatin in microparticles released from apoptotic cells [51]. DNase1 along with DNase1L3 are essential for disassembly of NETs [50,52].

The subsequent fate of such ecDNA in recipients’ blood is still unknown. DNase activity is one possible pathway of degradation of ecDNA, but other mechanisms of clearance cannot be discounted. The clearance of ecDNA could also be achieved by renal excretion into the urine [53] or uptake by the liver and spleen followed by macrophagic degradation [54]. Y-chromosome-specific sequences were detected in the urine of women who had been transfused with blood from male donors [55]. Detailed information on the mechanisms associated with these processes is lacking and remains somewhat controversial.

**Figure 1:**
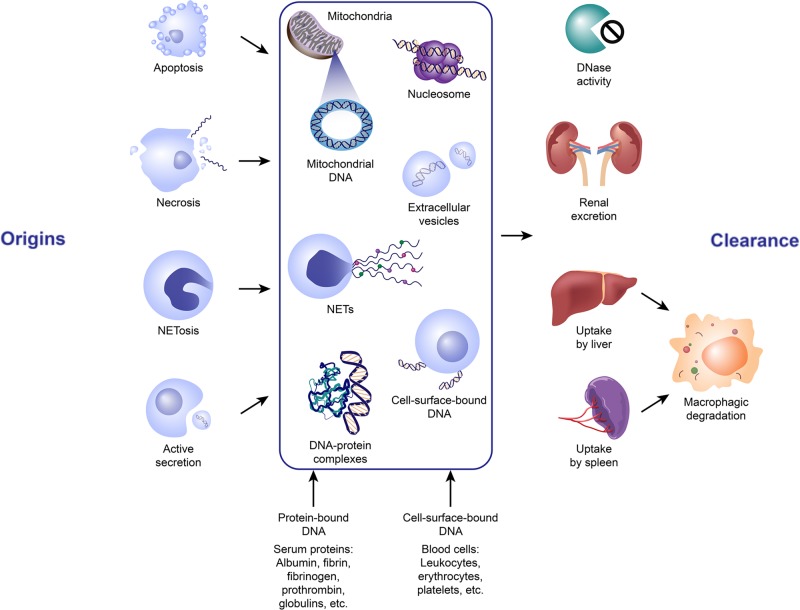
The potential origins and clearance of extracellular DNA in blood products ecDNA in blood exists in a number of forms, namely histone/DNA complexes or nucleosomes, extracellular vesicles packed DNA, cell-surface-bund DNA, DNA-protein complex, NETs and etc. It can be liberated from the blood cells via different mechanisms, most prominently apoptosis, necrosis, and active secretion, although other forms of cell death and clearance may contribute. Mitochondrial DNA can also be released by these mechanisms. Elimination of ecDNA could be achieved by DNase degradation, renal excretion into the urine or uptake by the liver and spleen followed by macrophagic degradation. Abbreviations: NETs, neutrophil extracellular traps.

A combination of DNase degradation, renal clearance, and uptake by the liver and spleen are likely to play a role in the clearance of ecDNA in transfusion recipients. Whether the ecDNA is complexed with lipid/proteins or nucleosomes, or is encapsulated within membrane-enclosed particles may influence the ability of DNases to clear ecDNA [4,56–58]. It was shown that DNA bound to nucleosomes can be protected from nuclease cleavage during apoptosis [56,57]. The ecDNA contained in EVs was also shown to be protected from degradation [4,58]. And the clearance of ecDNA may vary with the physiological state of the recipients [59]. Moreover, ecDNA can be recognized by various cell-surface DNA-binding proteins and can be transported into cells for possible degradation to mononucleotides or for transportation into the nucleus [60]. Therefore, the rate of ecDNA uptake by different cells may also affect the rate of its clearance. The potential origins and clearance of ecDNA in blood products are explained and summarized in Figure 1.

## Particular structures relevant to ecDNA in blood products

Depending upon different mechanisms of release, ecDNA is relevant to different complex structures; particulate structures such as EVs or macromolecular structures such as NETs and other less relevant structures that will not be detailed here [3].

### EVs

The first discovery of EVs was in 1964 when Chargaff and West, identified ´subcellular factors’ in cell-free plasma and showed that these factors played a role in blood clotting [61,62]. In 1967, Wolf [63] confirmed the presence of these subcellular factors using electron microscopy when he was studying the ´platelet dust’ that was known to be shed by platelets during storage [64]. Activated or apoptotic cells and platelets in blood products can shed small membrane vesicles, called EVs which are generally identified as MPs in transfusion research [62]. In particular, MPs derived from stored RBCs have been shown to contribute to neutrophil priming and activation, thereby enhancing the inflammatory response observed in patients who receive older RBCUs during transfusion [65].

Recently, the immunomodulatory potential of EVs in blood products has emerged as an important focus of studies in transfusion medicine. Based on the current knowledge, it has been suggested that EVs in stored blood are associated with a number of adverse outcomes such as neutrophil activation and the promotion of an inflammatory response in the recipients [41,65–67]. A study showed that EVs accumulating in RBC products during storage contribute to a strong inflammatory host response in recipients, which depends both on the number of EVs as well as on changes in the EVs related to storage [41]. Some studies suggested that platelet-derived EVs, such as those that convey mitochondrial DAMPs, may be a useful biomarker for the prediction of the potential risk of adverse transfusion reactions [68].

### NETs

NET formation, or ´NETosis’, was first described by Brinkmann et al. [36] in 2004. It occurs when neutrophils are activated by pathogen agents or under particular conditions: NETosis leads to chromatin decondensation, lysis of cell and nuclear membranes, and finally the release of NETs. The principal function of the NETs is believed to be to entrap and kill circulating pathogens.

The composition of NETs was initially widely believed to be predominantly nucDNA; however, under specific stimulatory conditions, NETs composed exclusively of mitDNA were demonstrated [69]. The emerging body of evidence suggests that NETs can indeed be composed exclusively or predominantly of mitDNA, which means that NETosis may represent a significant source of extracellular mitDNA in certain inflammatory conditions. In addition to the role of intracellular mitDNA in NET composition, mitDNA may also trigger NET formation as a DAMP after major trauma and with signaling mediated through a TLR9 dependent pathway [70]. Of note, Caudrillier et al. [71] reported that activated platelets induce the formation of NETs in transfusion-related acute lung injury (TRALI), which is the leading cause of death after transfusion therapy.

## Potential effects of ecDNA on transfusion

Over the past several decades, the effects of ecDNA on transfusion have rarely been investigated. A report published in 2018 pointed out that cell-free nucleic acids in blood products contained mainly double-stranded DNA (dsDNA), which has been shown to regulate genes of innate immune response [20]. The total ecDNA encompasses nucDNA and mitDNA. It was found that only mitDNA and bacterial DNA (bacDNA), increased neutrophil viability as a consequence of their activation [72]. In another report, mitDNA induced neutrophil matrix metalloproteinase 8 (MMP-8) and MMP-9 release, while nucDNA did not [73]. This evidence suggested that the extracellular mitDNA encompassed in ecDNA in transfusion may be immunological or proinflammatory. Here, we review the role of extracellular mitDNA in innate immune responses and its relationship with individual adverse reactions in transfusion. This section will also describe the potential role of transfusion in horizontal gene transfer (HGT).

### Extracellular mitDNA in innate immune responses

The particular DNA double-helix structure, the particular motifs of certain sequences and the molecular interactions are three factors at the origin of the stimulation of the immune response [74]. In effect, the exposure of cells of the innate immune system to dsDNA could provoke the activation of the genes of the innate immune response [20,74]. This stimulation is at the origin of a strong inflammatory response mediated by the secretion of cytokines. The abundant nucleic acid receptors in the cells play an important role in the innate immune system, which employs them in response to DNA within the hosts [75,76]. How exactly mitDNA may mediate their immunological role in transfusion is unknown, but different studies in some other areas of medicine rather than transfusion provided mechanistic insights. MitDNA has been shown to bind to Toll-like receptors (TLRs) or nucleotide oligomerization domain (NOD)-like receptors (NLRs) and more recently it has been shown to be linked with the stimulator of interferon genes (STING) pathway, thus providing distinct mechanisms potentially leading to immunological and inflammatory responses [77,78].

#### TLRs

Members of the TLR family are major pattern recognition receptors (PRRs) in cells [79], which are implicated in the innate immune response and are present in immune cells such as dendritic cells (DCs), neutrophils (PMNs), and macrophages (M∅) [80–83]. Zhang et al. [73,84] found that mitDNA activates neutrophil p38 mitogen-activated protein kinase (MAPK) through TLR9 with release of MMP-8, a proinflammatory cytokine, leading to severe inflammation in mouse lungs. Gu et al. [85] also found that intratracheal administration of mitDNA provokes lung inflammation through TLR9-p38 MAPK. In addition to MMP-8, mitDNA has been reported to trigger the activation of the nuclear factor kappa B (NFκB) pathway via TLR9 [86], resulting in up-regulation of proinflammatory cytokine production including TNF-α [87], IL-1β [88], and IL-6 [77].

#### NLRs

The NLRs are another major PRRs in the innate immune system. Of the NLRs, the NLR pyrin domain 3 (NLRP3) inflammasome is the most widely studied mainly due to its affinity for a wide variety of ligands [78]. The NLRP3 inflammasomes are targets of mitDNA, leading to the activation of caspase-1 in the inflammasome complex. Caspase-1 cleaves pro-IL-1β and pro-IL-18 into mature IL-1β and IL-18 [78], which is a potent pyrogen that elicits a strong proinflammatory response [89]. Shimada et al. [90] showed that it is the oxidized form of mitDNA that confers the inflammatogenic potential to mitDNA, which could directly bind NLRP3 to activate the inflammasome. Interestingly, the genetic deletion of NLRP3 and caspase-1 results in less mitDNA release [91]. Conversely, NLRP3 inflammasome formation releases mitDNA [91]. This suggests a positive feedback loop, in which activation of the NLRP3 inflammasome by oxidized mitDNA further promotes mitDNA release.

#### STING pathway

What’s more, mitDNA has the ability to stimulate the innate immune system through stimulation of the interferon genes (STING) pathway, resulting in interferon (IFN) release. The STING pathway was recently mechanistically dissected to reveal an intricate relationship demonstrating how mitDNA triggers interferon release [92]. The study showed that through the depletion of mitochondrial transcription factor A (TFAM), mitDNA stability was disturbed, causing enlargement of the mitochondrial nucleoid. Subsequently, fragmented mitDNA was released, activating peri-mitochondrial cyclic GMP-AMP synthase (cGAS) causing increased cGAMP formation. The second messenger cGAMP then activates the endoplasmic-reticulum-bound STING pathway which ultimately activates TANK-binding kinase 1 (TBK1) and results in IFN I and other interferon-stimulated genes [92].

**Figure 2:**
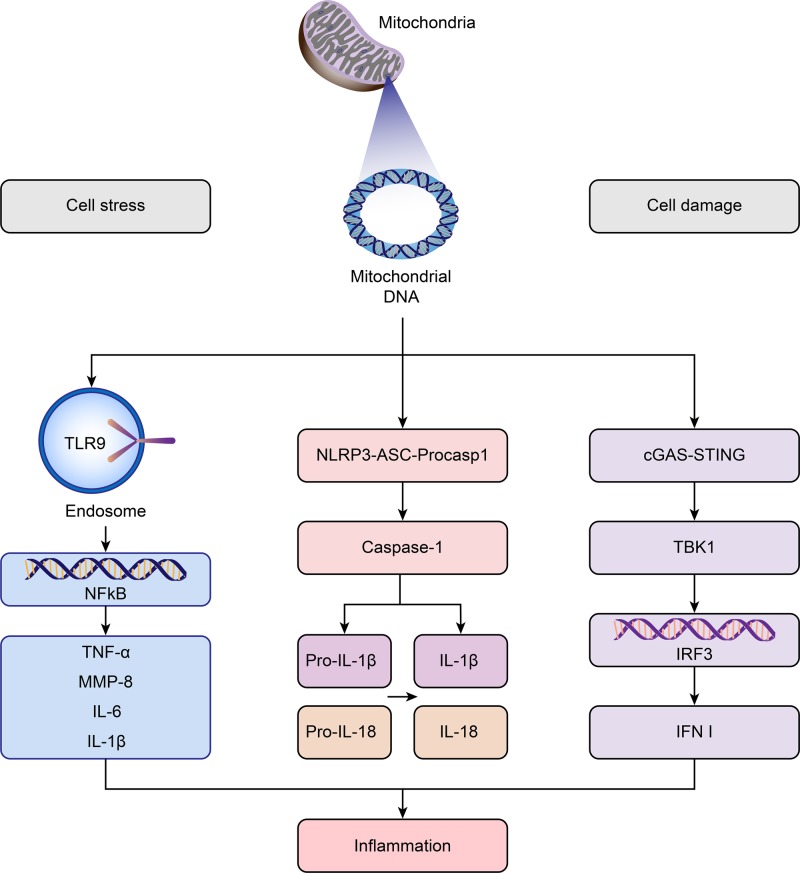
Innate immune pathways activated by mitochondrial DNA Upon cell stress or cell damage, mtDNA escaped from mitochondria engages in the activation of multiple innate immune pathways, including mitDNA–TLR9–NFκB axis, mitDNA–NLRP3–caspase1 pathway and mitDNA–cGAS–cGAMP–STING signaling, thereby igniting inflammation. Abbreviations: IFN, interferon; IRF, interferon regulatory factor; MMP, matrix metalloproteinase; NF-κB, nuclear transcription factor kappa B; NLRP3, Nod-like receptor pyrin domain containing 3; TBK1, TANK-binding kinase 1; TLR9, Toll-like receptor 9; TNF, tumor necrosis factor.

To conclude, mitDNA participates in a variety of innate immune pathways, including the mitDNA–TLR9–NFκB axis, mitDNA–NLRP3–caspase1 pathway and mitDNA–cGAS–cGAMP–STING signaling (as summarized in Figure 2). The extracellular mitDNA appears to be a potent danger signal that could be recognized by the innate immune system and modulate the inflammatory response [93].

### Extracellular mitDNA and adverse transfusion reactions

The establishment of strict procedures to avoid the transfusion of microbial components has greatly reduced the transmission of infections in recipients. However, sterile inflammation and organ injury in transfused recipients still occur in the absence of any apparent infectious agents [94]. TRALI is the leading cause of transfusion-related death and is initiated by soluble mediators in plasma [95]. Nonhemolytic transfusion reactions (NHTRs) are more frequent [96,97]. In addition, the transfusion of the plasma fraction (without cells or platelets) is sufficient to trigger these reactions [98].

Extracellular mitDNA falls into the category of DAMPs, which are known immune mediators associated with inflammation [22,23]. Studies have shown the potential contribution of extracellular mitDNA to adverse transfusion reactions [14,18,19,42]. Boudreau et al. [42] quantified mitDNA in the extracellular milieu of PCs that has induced adverse reactions and compared the levels to those within samples that were transfused without incidents. Interestingly, they confirmed that significantly higher levels of extracellular mitDNA correlated with adverse reactions [42]. Yasui et al. [18] further confirmed that elevated levels of mitDNA were present in PCs that induced NHTRs in platelet transfusion. Cognasse et al. [15] found that extracellular mitDNA did not correlate with cytokine levels and might be an independent risk factor in PC transfusion-linked inflammation.

TRALI is defined as new acute lung injury that develops during or within 6 h of receiving a transfusion of any blood product and progresses to a non-resolving severe respiratory failure such as acute respiratory distress syndrome (ARDS) [99]. Simmons et al. [19] found that the levels of mitDNA present in FFP and PCs correlate well with the levels of mitDNA in the serum of post-transfusion patients and are associated with a higher risk of ARDS. Mitochondrial DAMPs, including mitDNA, have been shown to potentiate inflammatory lung injury when introduced into healthy rats in a landmark paper by Zhang et al. [84]. In addition, mitDNA DAMPs could increase the EC permeability that was observed in acute lung injury and ARDS [100].

**Figure 3:**
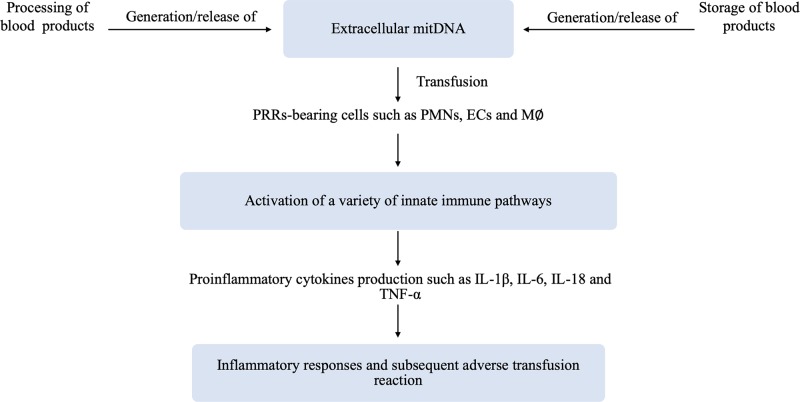
Working hypothesis of the involvement of extracellular mitDNA in the adverse transfusion reaction Briefly, the released/generated extracellular mitDNA in blood products during processing and storage time, which can be sensed by PRR-bearing cells of recipients (i.e. PMNs, ECs and M∅), is capable of activating a variety of innate immune pathways. Subsequent production of cytokines (i.e. IL-1β, IL-6, IL-18, and TNF-α) together with other proinflammatory mediator substances, thereby inducing inflammatory responses leading to adverse transfusion reactions in recipients. Abbreviations: EC, endothelial cell; INF, interferon; M∅, macrophages; PMN, neutrophil; PRR, pattern recognition receptor; TNF, tumor necrosis factor.

Extracellular mitDNA DAMPs present in transfusion products may act as a potential effector of TRALI, as hypothesized by Lee et al. [14]. According to current evidence as mentioned before, the working hypothesis of the involvement of extracellular mitDNA in the adverse transfusion reaction is summarized in Figure 3.

### The potential role of transfusion in HGT

During the 1950s to 1970s, blood transfusion experiments were found to alter the hereditary traits of the offspring in poultry [101–103]. As early as 1871, Galton [104] carried out intervarietal blood transfusion experiments among rabbits, but failed to induce heritable changes. Later, Sopikov [103] re-established the use of this method to analyse the effect of blood transfusion on hereditary traits: repeated transfusion of the blood of Black Australorp roosters to White Leghorn hens, and subsequent mating of these hens with White Leghorn roosters yielded progeny with modified inheritance. In exchanged donor and recipient roles, similar results were obtained [103]. Afterward, Sopikov’s observations were confirmed not only by many Soviet researchers [105,106], but also by investigators in other countries [107,108].

Subsequently, increasing evidence that DNA injection could induce heritable changes emerged [109,110]. For instance, DNA extracted from the Khaki Campbell was exclusively used to induce heritable changes in the Pekin duck in the study of Benoit et al. [111] Hereditary modifications of morphological characteristics in ducks as a result of the injection of DNA and RNA from other breeds of ducks were also reported. It is clearly possible that when DNA-rich avian blood cells are transfused to other members of the same species, the transferred DNA can be expressed. Currently, many researchers have detected ecDNA in the plasma of donor-derived blood products, as listed before in Table 1.

Landman [112] suggested that the genes of organisms could be divided into two groups: most were inherited ‘vertically’ from ancestors, but some were acquired ‘horizontally’. HGT is defined as the transfer of genes between organisms in a fashion other than that, which is found through traditional reproduction [113]. The transfer of biological sources, including blood transfusion products, from donor to recipient will likely result in the presence of donor ecDNA in the recipient’s blood circulation. This raises concern over the potential of ecDNA to transfer the genetic or epigenetic information of clinical risk factors (genetically related illness, mutations or pharmaceutically induced adverse effects) from donors to recipients.

Stroun and Anker [114] have been suggested that nucleic acids are released by living cells and circulate throughout the whole organism. Newly synthesized, actively released ecDNA can translocate to neighbouring and remote parts of the body, enter cells and alter their biology [115,116]. Genometastasis experiments have shown that ecDNA in the plasma of cancer patients can indeed transfer oncogenic information to susceptible cells [24]. Thierry et al. [3] illustrated that many structures carrying ecDNA could be involved in this genometastasis. However, this implies that ecDNA in the plasma of blood transfusion products might be capable of transferring an activated human oncogene to recipients during blood transfusions. In addition, many pharmaceutical and botanical compounds have been found to induce epigenetic alterations [117–119]. It is, therefore, possible that the ecDNA in donors using medication can contain these pharmaceutically induced epigenetic alterations and that these alterations can be transferred to recipients during blood transfusions.

Moreover, it was shown that microchimerism might be related to real or potential health implications in autoimmune diseases, graft-versus-host reactions, and transfusion complications [120]. Microchimerism refers to a small number of cells or DNA in one individual that is derived from another genetically distinct individual [121]. Regarding blood transfusion, microchimerism from non-leukoreduced cellular blood products has been found to persist from months to years after transfusion in traumatically injured patients [122]. Transfusion-associated microchimerism appears to be an identified complication of blood transfusion [123,124]. Whether patients with transfusion-associated microchimerism have consequent adverse health effects, such as transfusion associated graft-versus-host disease, is still under investigation.

## Conclusion

Blood transfusion is the intravascular transfer of blood products into a recipient. A certain amount of DNA encompassed mitDNA was demonstrated to be present in the extracellular milieu of blood products for transfusion. This raises the question of whether such ecDNA carries a risk. We provide a comprehensive review of the potential origins, clearance mechanisms and relevant structures of ecDNA in blood products in order to familiarize researchers with previous works and to show that there are still many unanswered questions relating to the nature and biological functions of ecDNA in blood products. Our review shows that ecDNA especially mitDNA participates in a variety of innate immune pathways and relevant to some adverse transfusion reactions. In addition, it should be concerned that ecDNA might be capable of transferring some genetic or epigenetic information of clinical risk factors from donors to recipients during blood transfusion. Notably, methods of isolation and quantification of ecDNA are crucial when analysing data from different reports. At this time, there is a lack of uniformity in the methods of ecDNA extraction and quantification, which need to be standardized. What’s more, the detailed clearance mechanisms of these ecDNA in recipients administrated via transfusion also require further research, including (i) the activity of DNase, (ii) the rate of renal excretion into urine, and (iii) the rate of uptake by the liver and spleen. However, this remains an underexplored field in transfusion, and more insights will likely emerge in the near future. Hopefully, the articles presented herein will stimulate researchers to give serious consideration to the potential harmful effects of ecDNA, especially extracellular mitDNA, on transfusion.

## Abbreviations

ARDS: acute respiratory distress syndrome
bacDNA: bacterial DNA
cfDNA: cell-free DNA
cGAS: cyclic GMP-AMP synthase
DAMP: damage-associated molecular pattern
DC: dendritic cell
dsDNA: double-stranded DNA
ecDNA: extracellular DNA
EV: extracellular vehicle
FFP: fresh frozen plasma
HGT: horizontal gene transfer
IFN: interferon
IL-1β: interleukin-1β
IL-6: interleukin-6
IL-18: interleukin-18
M∅: macrophages
MAPK: mitogen-activated protein kinase
mitDNA: mitochondrial DNA
MMP-8/9: metalloproteinase-8/9
MP: microparticle
NET: neutrophil extracellular trap
NF-κB: nuclear factor kappa B
NHTR: nonhemolytic transfusion reaction
NLRP3: NLR pyrin domain 3
NLR: nucleotide oligomerization domain (NOD)-like receptor
nucDNA: nuclear DNA
PC: platelet concentrate
PMN: neutrophil
PRR: pattern recognition receptor
RBC: red blood cell
RBCU: red blood cell unit
sPLA2-IIA: secretory phospholipase A2 IIA
STING: stimulator of interferon genes
TBK1: TANK-binding kinase 1
TFAM: mitochondrial transcription factor A
TLR: toll-like receptor
TNF-α: tumor necrosis factor α
TRALI: transfusion-related acute lung injury

## References

[1] Mandel P. and Métais P. (1948) Les acides nucléiques du plasma sanguin chez l'homme. C. R. Seances Soc. Biol. Fil. 142, 241–243 18875018

[2] Tamkovich S.N., Bryzgunova O.E., Rykova E.Y., Permyakova V.I., Vlassov V.V. and Laktionov P.P. (2005) Circulating nucleic acids in blood of healthy male and female donors. Clin. Chem. 51, 1317–1319 10.1373/clinchem.2004.045062 15976134

[3] Thierry A.R., El Messaoudi S., Gahan P.B., Anker P. and Stroun M. (2016) Origins, structures, and functions of circulating DNA in oncology. Cancer Metastasis Rev. 35, 347–376 10.1007/s10555-016-9629-x 27392603 PMC5035665

[4] Fernando M.R., Jiang C., Krzyzanowski G.D. and Ryan W.L. (2017) New evidence that a large proportion of human blood plasma cell-free DNA is localized in exosomes. PLoS ONE 12, e0183915 10.1371/journal.pone.0183915 28850588 PMC5574584

[5] Kustanovich A., Schwartz R., Peretz T. and Grinshpun A. (2019) Life and death of circulating cell-free DNA. Cancer Biol. Ther. 20, 1057–1067 10.1080/15384047.2019.1598759 30990132 PMC6606043

[6] Dwivedi D.J., Toltl L.J., Swystun L.L., Pogue J., Liaw K.-L., Weitz J.I. et al. (2012) Prognostic utility and characterization of cell-free DNA in patients with severe sepsis. Crit. Care 16, R151 10.1186/cc11466 22889177 PMC3580740

[7] Lo Y.M.D., Rainer T.H., Chan L.Y.S., Hjelm N.M. and Cocks R.A. (2000) Plasma DNA as a prognostic marker in trauma patients. Clin. Chem. 46, 319–323 10.1093/clinchem/46.3.319 10702517

[8] Rainer T.H., Wong L.K.S., Lam W., Yuen E., Lam N.Y.L., Metreweli C. et al. (2003) Prognostic use of circulating plasma nucleic acid concentrations in patients with acute stroke. Clin. Chem. 49, 562–569 10.1373/49.4.562 12651807

[9] Chang C.P.Y., Chia R.-H., Wu T.-L., Tsao K.-C., Sun C.-F. and Wu J.T. (2003) Elevated cell-free serum DNA detected in patients with myocardial infarction. Clin. Chim. Acta 327, 95–101 10.1016/S0009-8981(02)00337-6 12482623

[10] Steinman C.R. (1984) Circulating DNA in systemic lupus erythematosus. Isolation and characterization. J. Clin. Invest. 73, 832–841 10.1172/JCI111278 6323528 PMC425087

[11] Duxbury M., Jezuit M., Letwin B. and Wright J. (1995) DNA in plasma of human blood for transfusion. Biologicals 23, 229–231 10.1006/biol.1995.0038 8527123

[12] Dijkstra-Tiekstra M.J., Pietersz R.N.I., Reesink H.W. and van der Schoot C.E. (2004) Influence of cell-free DNA in plasma on real-time polymerase chain reaction for determination of residual leucocytes in platelet concentrates. Vox Sang 86, 130–135 10.1111/j.0042-9007.2004.00402.x 15023183

[13] Ivancic-Jelecki J., Brgles M., Santak M. and Forcic D. (2009) Isolation of cell-free DNA from plasma by chromatography on short monolithic columns and quantification of non-apoptotic fragments by real-time polymerase chain reaction. J. Chromatogr. A 1216, 2717–2724 10.1016/j.chroma.2008.10.087 19007935

[14] Lee Y.L., King M.B., Gonzalez R.P., Brevard S.B., Frotan M.A., Gillespie M.N. et al. (2014) Blood transfusion products contain mitochondrial DNA damage-associated molecular patterns: a potential effector of transfusion-related acute lung injury. J. Surg. Res. 191, 286–289 10.1016/j.jss.2014.06.003 25039013 PMC4308969

[15] Cognasse F., Aloui C., Anh Nguyen K., Hamzeh-Cognasse H., Fagan J., Arthaud C.A. et al. (2016) Platelet components associated with adverse reactions: predictive value of mitochondrial DNA relative to biological response modifiers. Transfusion 56, 497–504 10.1111/trf.13373 26446055

[16] Shih A.W., Bhagirath V.C., Heddle N.M., Acker J.P., Liu Y., Eikelboom J.W. et al. (2016) Quantification of cell-free DNA in red blood cell units in different whole blood processing methods. J. Blood Transfus. 2016, 9316385 10.1155/2016/9316385 27774338 PMC5059535

[17] Bakkour S., Acker J.P., Chafets D.M., Inglis H.C., Norris P.J., Lee T.H. et al. (2016) Manufacturing method affects mitochondrial DNA release and extracellular vesicle composition in stored red blood cells. Vox Sang 111, 22–32 10.1111/vox.12390 26918437

[18] Yasui K., Matsuyama N., Kuroishi A., Tani Y., Furuta R.A. and Hirayama F. (2016) Mitochondrial damage-associated molecular patterns as potential proinflammatory mediators in post-platelet transfusion adverse effects. Transfusion 56, 1201–1212 10.1111/trf.13535 26920340

[19] Simmons J.D., Lee Y.-L.L., Pastukh V.M., Capley G., Muscat C.A., Muscat D.C. et al. (2017) Potential contribution of mitochondrial DNA damage associated molecular patterns in transfusion products to the development of acute respiratory distress syndrome after multiple transfusions. J. Trauma Acute Care Surg. 82, 1023–1029 10.1097/TA.0000000000001421 28301393 PMC5472063

[20] Waldvogel Abramowski S., Tirefort D., Lau P., Guichebaron A., Taleb S., Modoux C. et al. (2018) Cell-free nucleic acids are present in blood products and regulate genes of innate immune response. Transfusion 58, 1671–1681 10.1111/trf.14613 29664127

[21] Krysko D.V., Agostinis P., Krysko O., Garg A.D., Bachert C., Lambrecht B.N. et al. (2011) Emerging role of damage-associated molecular patterns derived from mitochondria in inflammation. Trends Immunol 32, 157–164 10.1016/j.it.2011.01.005 21334975

[22] Seong S.Y. and Matzinger P. (2004) Hydrophobicity: an ancient damage-associated molecular pattern that initiates innate immune responses. Nat. Rev. Immunol. 4, 469–478 10.1038/nri1372 15173835

[23] Schaefer L. (2014) Complexity of danger: the diverse nature of damage-associated molecular patterns. J. Biol. Chem. 289, 35237–35245 10.1074/jbc.R114.619304 25391648 PMC4271212

[24] Garcia-Olmo D.C., Dominguez C., Garcia-Arranz M., Anker P., Stroun M., Garcia-Verdugo J.M. et al. (2010) Cell-free nucleic acids circulating in the plasma of colorectal cancer patients induce the oncogenic transformation of susceptible cultured cells. Cancer Res 70, 560–567 10.1158/0008-5472.CAN-09-3513 20068178

[25] Gray M.W. (2012) Mitochondrial evolution. Cold Spring Harb. Perspect. Biol. 4, a011403 10.1101/cshperspect.a011403 22952398 PMC3428767

[26] Zimmer C. (2009) Origins. On the origin of eukaryotes. Science 325, 666–668 10.1126/science.325_666 19661396

[27] Hochhauser D. (2000) Relevance of mitochondrial DNA in cancer. Lancet 356, 181–182 10.1016/S0140-6736(00)02475-2 10963192

[28] Aucamp J., Bronkhorst A.J., Badenhorst C.P.S. and Pretorius P.J. (2018) The diverse origins of circulating cell-free DNA in the human body: a critical re-evaluation of the literature. Biol. Rev. 93, 1649–1683 10.1111/brv.12413 29654714

[29] Jahr S., Hentze H., Englisch S., Hardt D., Fackelmayer F.O., Hesch R.-D. et al. (2001) DNA fragments in the blood plasma of cancer patients: quantitations and evidence for their origin from apoptotic and necrotic cells. Cancer Res 61, 1659–1665 11245480

[30] Suzuki N., Kamataki A., Yamaki J. and Homma Y. (2008) Characterization of circulating DNA in healthy human plasma. Clin. Chim. Acta 387, 55–58 10.1016/j.cca.2007.09.001 17916343

[31] Bronkhorst A.J., Ungerer V. and Holdenrieder S. (2019) The emerging role of cell-free DNA as a molecular marker for cancer management. Biomol. Detect. Quantif. 17, 100087 10.1016/j.bdq.2019.100087 30923679 PMC6425120

[32] Stroun M. and Anker P. (1972) *In vitro* synthesis of DNA spontaneously released by bacteria or frog auricles. Biochimie 54, 1443–1452 10.1016/S0300-9084(72)80086-5 4196378

[33] Borenstein S. and Ephrati-Elizur E. (1969) Spontaneous release of DNA in sequential genetic order by *Bacillus subtilis*. J. Mol. Biol. 45, 137–152 10.1016/0022-2836(69)90216-2 4981003

[34] Ermakov A.V., Kostyuk S.V., Konkova M.S., Egolina N.A., Malinovskaya E.M. and Veiko N.N. (2008) Extracellular DNA fragments. Ann. N. Y. Acad. Sci. 1137, 41–46 10.1196/annals.1448.024 18837923

[35] Anker P., Stroun M. and Maurice P.A. (1975) Spontaneous release of DNA by human blood lymphocytes as shown in an *in vitro* system. Cancer Res 35, 2375–2382 1149042

[36] Brinkmann V., Reichard U., Goosmann C., Fauler B., Uhlemann Y., Weiss D.S. et al. (2004) Neutrophil extracellular traps kill bacteria. Science 303, 1532–1535 10.1126/science.1092385 15001782

[37] Keshari R.S., Jyoti A., Kumar S., Dubey M., Verma A., Srinag B.S. et al. (2012) Neutrophil extracellular traps contain mitochondrial as well as nuclear DNA and exhibit inflammatory potential. Cytometry A 81, 238–247 10.1002/cyto.a.21178 22170804

[38] Yousefi S., Mihalache C., Kozlowski E., Schmid I. and Simon H.U. (2009) Viable neutrophils release mitochondrial DNA to form neutrophil extracellular traps. Cell Death Differ 16, 1438–1444 10.1038/cdd.2009.96 19609275

[39] Pisetsky D.S., Gauley J. and Ullal A.J. (2011) Microparticles as a source of extracellular DNA. Immunol. Res. 49, 227–234 10.1007/s12026-010-8184-8 21132466 PMC3724471

[40] Marcoux G., Duchez A.C., Rousseau M., Levesque T., Boudreau L.H., Thibault L. et al. (2017) Microparticle and mitochondrial release during extended storage of different types of platelet concentrates. Platelets 28, 272–280 10.1080/09537104.2016.1218455 27681879

[41] Straat M., Boing A.N., Tuip-De Boer A., Nieuwland R. and Juffermans N.P. (2016) Extracellular vesicles from red blood cell products induce a strong pro-inflammatory host response, dependent on both numbers and storage duration. Transfus. Med. Hemother. 43, 302–305 10.1159/000442681 27721707 PMC5040938

[42] Boudreau L.H., Duchez A.-C., Cloutier N., Soulet D., Martin N., Bollinger J. et al. (2014) Platelets release mitochondria serving as substrate for bactericidal group IIA-secreted phospholipase A2 to promote inflammation. Blood 124, 2173–2183 10.1182/blood-2014-05-573543 25082876 PMC4260364

[43] Tan E.M., Schur P.H., Carr R.I. and Kunkel H.G. (1966) Deoxybonucleic acid (DNA) and antibodies to DNA in the serum of patients with systemic lupus erythematosus. J. Clin. Invest. 45, 1732–1740 10.1172/JCI105479 4959277 PMC292857

[44] Raptis L. and Menard H.A. (1980) Quantitation and characterization of plasma DNA in normals and patients with systemic lupus erythematosus. J. Clin. Invest. 66, 1391–1399 10.1172/JCI109992 7440721 PMC371625

[45] Steinman C.R. (1975) Free DNA in serum and plasma from normal adults. J. Clin. Invest. 56, 512–515 10.1172/JCI108118 1150882 PMC436612

[46] Volkova Z.I., Solov'ev G. and Folomeeva O.M. (1980) Isolation and characteristics of the DNA of donor and systemic lupus erythematosus patient blood plasma. Biull. Eksp. Biol. Med. 89, 689–691 10.1007/BF00836247 7397359

[47] Stroun M., Anker P., Lyautey J., Lederrey C. and Maurice P.A. (1987) Isolation and characterization of DNA from the plasma of cancer patients. Eur. J. Cancer Clin. Oncol. 23, 707–712 10.1016/0277-5379(87)90266-5 3653190

[48] Soni C. and Reizis B. (2019) Self-DNA at the epicenter of SLE: immunogenic forms, regulation, and effects. Front. Immunol. 10, 1601–1601 10.3389/fimmu.2019.01601 31354738 PMC6637313

[49] Napirei M., Gultekin A., Kloeckl T., Moroy T., Frostegard J. and Mannherz H.G. (2006) Systemic lupus-erythematosus: deoxyribonuclease 1 in necrotic chromatin disposal. Int. J. Biochem. Cell Biol. 38, 297–306 10.1016/j.biocel.2005.10.023 16352456

[50] Napirei M., Ludwig S., Mezrhab J., Klöckl T. and Mannherz H.G. (2009) Murine serum nucleases-contrasting effects of plasmin and heparin on the activities of DNase1 and DNase1-like 3 (DNase1l3). FEBS J. 276, 1059–1073 10.1111/j.1742-4658.2008.06849.x 19154352

[51] Sisirak V., Sally B., D'Agati V., Martinez-Ortiz W., Ozcakar Z.B., David J. et al. (2016) Digestion of chromatin in apoptotic cell microparticles prevents autoimmunity. Cell 166, 88–101 10.1016/j.cell.2016.05.034 27293190 PMC5030815

[52] Jiménez-Alcázar M., Rangaswamy C., Panda R., Bitterling J., Simsek Y.J., Long A.T. et al. (2017) Host DNases prevent vascular occlusion by neutrophil extracellular traps. Science 358, 1202–1206 10.1126/science.aam8897 29191910

[53] Yu S.C., Lee S.W., Jiang P., Leung T.Y., Chan K.C., Chiu R.W. et al. (2013) High-resolution profiling of fetal DNA clearance from maternal plasma by massively parallel sequencing. Clin. Chem. 59, 1228–1237 10.1373/clinchem.2013.203679 23603797

[54] Chused T.M., Steinberg A.D. and Talal N. (1972) The clearance and localization of nucleic acids by New Zealand and normal mice. Clin. Exp. Immunol. 12, 465–476 4650369 PMC1553612

[55] Botezatu I., Serdyuk O., Potapova G., Shelepov V., Alechina R., Molyaka Y. et al. (2000) Genetic analysis of DNA excreted in urine: a new approach for detecting specific genomic DNA sequences from cells dying in an organism. Clin. Chem. 46, 1078–1084 10.1093/clinchem/46.8.1078 10926886

[56] Thierry A.R., Rabinovich P., Peng B., Mahan L.C., Bryant J.L. and Gallo R.C. (1997) Characterization of liposome-mediated gene delivery: expression, stability and pharmacokinetics of plasmid DNA. Gene Ther. 4, 226–237 10.1038/sj.gt.3300350 9135736

[57] Ma X., Zhu L., Wu X., Bao H., Wang X., Chang Z. et al. (2017) Cell-free DNA provides a good representation of the tumor genome despite its biased fragmentation patterns. PLoS ONE 12, e0169231 10.1371/journal.pone.0169231 28046008 PMC5207727

[58] Contreras-Naranjo J.C., Wu H.J. and Ugaz V.M. (2017) Microfluidics for exosome isolation and analysis: enabling liquid biopsy for personalized medicine. Lab. Chip. 17, 3558–3577 10.1039/C7LC00592J 28832692 PMC5656537

[59] Leung F., Kulasingam V., Diamandis E.P., Hoon D.S.B., Kinzler K., Pantel K. et al. (2016) Circulating tumor DNA as a cancer biomarker: fact or fiction? Clin. Chem. 62, 1054–1060 10.1373/clinchem.2016.260331 27259816 PMC5326709

[60] Chelobanov B.P., Laktionov P.P. and Vlasov V.V. (2006) Proteins involved in binding and cellular uptake of nucleic acids. Biochemistry (Mosc) 71, 583–596 10.1134/S0006297906060010 16827649

[61] Chargaff E. and West R. (1946) The biological significance of the thromboplastic protein of blood. J. Biol. Chem. 166, 189–197 20273687

[62] van der Pol E., Boing A.N., Harrison P., Sturk A. and Nieuwland R. (2012) Classification, functions, and clinical relevance of extracellular vesicles. Pharmacol. Rev. 64, 676–705 10.1124/pr.112.005983 22722893

[63] Wolf P. (1967) The nature and significance of platelet products in human plasma. Br. J. Haematol. 13, 269–288 10.1111/j.1365-2141.1967.tb08741.x 6025241

[64] de Vooght K.M., Lau C., de Laat P.P., van Wijk R., van Solinge W.W. and Schiffelers R.M. (2013) Extracellular vesicles in the circulation: are erythrocyte microvesicles a confounder in the plasma haemoglobin assay? Biochem. Soc. Trans. 41, 288–292 10.1042/BST20120254 23356299

[65] Belizaire R.M., Prakash P.S., Richter J.R., Robinson B.R., Edwards M.J., Caldwell C.C. et al. (2012) Microparticles from stored red blood cells activate neutrophils and cause lung injury after hemorrhage and resuscitation. J. Am. Coll. Surg. 214, 648–655 10.1016/j.jamcollsurg.2011.12.032 22342784 PMC4034387

[66] Cardo L.J., Wilder D. and Salata J. (2008) Neutrophil priming, caused by cell membranes and microvesicles in packed red blood cell units, is abrogated by leukocyte depletion at collection. Transfus. Apher. Sci. 38, 117–125 10.1016/j.transci.2008.01.004 18343726

[67] Almizraq R.J., Seghatchian J. and Acker J.P. (2016) Extracellular vesicles in transfusion-related immunomodulation and the role of blood component manufacturing. Transfus. Apher. Sci. 55, 281–291 10.1016/j.transci.2016.10.018 27865649

[68] Marcoux G., Magron A., Sut C., Laroche A., Laradi S., Hamzeh-Cognasse H. et al. (2019) Platelet-derived extracellular vesicles convey mitochondrial DAMPs in platelet concentrates and their levels are associated with adverse reactions. Transfusion 59, 2403–2414 10.1111/trf.15300 30973972

[69] Yousefi S., Gold J.A., Andina N., Lee J.J., Kelly A.M., Kozlowski E. et al. (2008) Catapult-like release of mitochondrial DNA by eosinophils contributes to antibacterial defense. Nat. Med. 14, 949–953 10.1038/nm.1855 18690244

[70] Pinti M., Cevenini E., Nasi M., de Biasi S., Salvioli S., Monti D. et al. (2014) Circulating mitochondrial DNA increases with age and is a familiar trait: implications for “inflamm-aging”. Eur. J. Immunol. 44, 1552–1562 10.1002/eji.201343921 24470107

[71] Caudrillier A., Kessenbrock K., Gilliss B.M., Nguyen J.X., Marques M.B., Monestier M. et al. (2012) Platelets induce neutrophil extracellular traps in transfusion-related acute lung injury. J. Clin. Invest. 122, 2661–2671 10.1172/JCI61303 22684106 PMC3386815

[72] Bhagirath V.C., Dwivedi D.J. and Liaw P.C. (2015) Comparison of the proinflammatory and procoagulant properties of nuclear, mitochondrial, and bacterial DNA. Shock 44, 265–271 10.1097/SHK.0000000000000397 25944792

[73] Zhang Q., Itagaki K. and Hauser C.J. (2010) Mitochondrial DNA is released by shock and activates neutrophils via p38 map kinase. Shock 34, 55–59 10.1097/SHK.0b013e3181cd8c08 19997055

[74] Pisetsky D.S. (2012) The origin and properties of extracellular DNA: from PAMP to DAMP. Clin. Immunol. 144, 32–40 10.1016/j.clim.2012.04.006 22659033 PMC3724456

[75] Kawasaki T., Kawai T. and Akira S. (2011) Recognition of nucleic acids by pattern-recognition receptors and its relevance in autoimmunity. Immunol. Rev. 243, 61–73 10.1111/j.1600-065X.2011.01048.x 21884167 PMC7165622

[76] Xiao T. (2009) Innate immune recognition of nucleic acids. Immunol. Res. 43, 98–108 10.1007/s12026-008-8053-x 18810334 PMC3318969

[77] Zhang J.Z., Liu Z., Liu J., Ren J.X. and Sun T.S. (2014) Mitochondrial DNA induces inflammation and increases TLR9/NF-kappaB expression in lung tissue. Int. J. Mol. Med. 33, 817–824 10.3892/ijmm.2014.1650 24535292 PMC3976143

[78] Gurung P., Lukens J.R. and Kanneganti T.D. (2015) Mitochondria: diversity in the regulation of the NLRP3 inflammasome. Trends Mol. Med. 21, 193–201 10.1016/j.molmed.2014.11.008 25500014 PMC4352396

[79] Newton K. and Dixit V.M. (2012) Signaling in innate immunity and inflammation. Cold Spring Harb. Perspect. Biol. 4, a006049 10.1101/cshperspect.a006049 22296764 PMC3282411

[80] Takeda K. and Akira S. (2005) Toll-like receptors in innate immunity. Int. Immunol. 17, 1–14 10.1093/intimm/dxh186 15585605

[81] Beg A.A. (2002) Endogenous ligands of Toll-like receptors: implications for regulating inflammatory and immune responses. Trends Immunol. 23, 509–512 10.1016/S1471-4906(02)02317-7 12401394

[82] Kawai T. and Akira S. (2010) The role of pattern-recognition receptors in innate immunity: update on Toll-like receptors. Nat. Immunol. 11, 373–384 10.1038/ni.1863 20404851

[83] Nigar S. and Shimosato T. (2019) Cooperation of oligodeoxynucleotides and synthetic molecules as enhanced immune modulators. Front. Nutr. 6, 140 10.3389/fnut.2019.00140 31508424 PMC6718720

[84] Zhang Q., Raoof M., Chen Y., Sumi Y., Sursal T., Junger W. et al. (2010) Circulating mitochondrial DAMPs cause inflammatory responses to injury. Nature 464, 104–107 10.1038/nature08780 20203610 PMC2843437

[85] Gu X., Wu G., Yao Y., Zeng J., Shi D., Lv T. et al. (2015) Intratracheal administration of mitochondrial DNA directly provokes lung inflammation through the TLR9-p38 MAPK pathway. Free Radic. Biol. Med. 83, 149–158 10.1016/j.freeradbiomed.2015.02.034 25772007

[86] Krychtiuk K.A., Ruhittel S., Hohensinner P.J., Koller L., Kaun C., Lenz M. et al. (2015) Mitochondrial DNA and toll-like receptor-9 are associated with mortality in critically ill patients. Crit. Care Med. 43, 2633–2641 10.1097/CCM.0000000000001311 26448617

[87] Julian M.W., Shao G., Vangundy Z.C., Papenfuss T.L. and Crouser E.D. (2013) Mitochondrial transcription factor A, an endogenous danger signal, promotes TNFalpha release via RAGE- and TLR9-responsive plasmacytoid dendritic cells. PLoS ONE 8, e72354 10.1371/journal.pone.0072354 23951313 PMC3741150

[88] Yu E.P. and Bennett M.R. (2014) Mitochondrial DNA damage and atherosclerosis. Trends Endocrinol. Metab. 25, 481–487 10.1016/j.tem.2014.06.008 25034130

[89] Dinarello C.A. (2009) Immunological and inflammatory functions of the interleukin-1 family. Annu. Rev. Immunol. 27, 519–550 10.1146/annurev.immunol.021908.132612 19302047

[90] Shimada K., Crother T.R., Karlin J., Dagvadorj J., Chiba N., Chen S. et al. (2012) Oxidized mitochondrial DNA activates the NLRP3 inflammasome during apoptosis. Immunity 36, 401–414 10.1016/j.immuni.2012.01.009 22342844 PMC3312986

[91] Nakahira K., Haspel J.A., Rathinam V.A., Lee S.J., Dolinay T., Lam H.C. et al. (2011) Autophagy proteins regulate innate immune responses by inhibiting the release of mitochondrial DNA mediated by the NALP3 inflammasome. Nat. Immunol. 12, 222–230 10.1038/ni.1980 21151103 PMC3079381

[92] West A.P., Khoury-Hanold W., Staron M., Tal M.C., Pineda C.M., Lang S.M. et al. (2015) Mitochondrial DNA stress primes the antiviral innate immune response. Nature 520, 553–557 10.1038/nature14156 25642965 PMC4409480

[93] Rock K.L., Latz E., Ontiveros F. and Kono H. (2010) The sterile inflammatory response. Annu. Rev. Immunol. 28, 321–342 10.1146/annurev-immunol-030409-101311 20307211 PMC4315152

[94] Garraud O., Sut C., Haddad A., Tariket S., Aloui C., Laradi S. et al. (2018) Transfusion-associated hazards: a revisit of their presentation. Transfus. Clin. Biol. 25, 118–135 10.1016/j.tracli.2018.03.002 29625790

[95] Semple J.W., McVey M.J., Kim M., Rebetz J., Kuebler W.M. and Kapur R. (2018) Targeting transfusion-related acute lung injury: the journey from basic science to novel therapies. Crit. Care Med. 46, e452–e458 10.1097/CCM.0000000000002989 29384784

[96] Heddle N.M. (1995) Febrile nonhemolytic transfusion reactions to platelets. Curr. Opin. Hematol. 2, 478–483 10.1097/00062752-199502060-00013 9372039

[97] Sahler J., Grimshaw K., Spinelli S.L., Refaai M.A., Phipps R.P. and Blumberg N. (2011) Platelet storage and transfusions: new concerns associated with an old therapy. Drug Discov. Today Dis. Mech. 8, e9–e14 10.1016/j.ddmec.2011.06.001 22662018 PMC3361759

[98] Heddle N.M., Klama L., Singer J., Richards C., Fedak P., Walker I. et al. (1994) The role of the plasma from platelet concentrates in transfusion reactions. N. Engl. J. Med. 331, 625–628 10.1056/NEJM199409083311001 8052271

[99] Marik P.E. and Corwin H.L. (2008) Acute lung injury following blood transfusion: expanding the definition. Crit. Care Med. 36, 3080–3084 10.1097/CCM.0b013e31818c3801 18824899

[100] Sun S., Sursal T., Adibnia Y., Zhao C., Zheng Y., Li H. et al. (2013) Mitochondrial DAMPs increase endothelial permeability through neutrophil dependent and independent pathways. PLoS ONE 8, e59989 10.1371/journal.pone.0059989 23527291 PMC3603956

[101] Golubev A.K. (1964) Variability in Fowls due to the Transfer of Blood and its Components, Kolos, Leningrad

[102] Stroun J., Stroun-Guttieres L., Rossi J. and Stroun M. (1962) Alteration of the color of the feathers in white Leghorn chickens by repeated injections of the blood of guinea hens. Observations on 5 generations. C. R. Hebd. Seances Acad. Sci. 255, 781–783 13917870

[103] Sopikov P.M. (1950) A new method of vegetative hybridisation in poultry by blood transfusion. Priroda 39, 66

[104] Galton F. (1871) I. Experiments in Pangenesis, by breeding from rabbits of a pure variety, into whose circulation blood taken from other varieties had previously been largely transfused. Proc. R. Soc. Lond. 19, 393–410

[105] Golubev A.K. (1966) Alteration of chicken heredity after transfusion of blood and blood components from other breeds. In The Proceedings of the 13th World's Poultry Congress (Duyunov E.A. and Kopylopylovskaya G., eds), pp. 129–135, USSR, Kiev

[106] Gromov A.M. (1870) Character of changes during haemohybridization in fowl with conservative and recessive plumage colour. The Proceedings of the 14th World's Poultry Congress, pp. 65–72, WPSA, Madrid

[107] Stroun J., Stroun-Guttieres L., Rossi J. and Stroun M. (1963) Transfer to the progeny of alterations induced in the white leghorn by repeated injections of heterologous blood. Arch. Des Sci. 16, 247–262

[108] Leroy P., Vendrely R., Benoit J. and Vendrely C. (1966) Divergences observed in the descendants of Rhode Island red chickens M-44 after injections of specifically different bloods or fractions of blood. In The Proceedings of the 13th World's Poultry Congress (Duyunov E.A. and Kopylopylovskaya G., eds), pp. 106–109, USSR, Kiev

[109] Tsukamoto M., Ochiya T., Yoshida S., Sugimura T. and Terada M. (1995) Gene transfer and expression in progeny after intravenous DNA injection into pregnant mice. Nat. Genet. 9, 243–248 10.1038/ng0395-243 7773286

[110] Schubbert R., Hohlweg U., Renz D. and Doerfler W. (1998) On the fate of orally ingested foreign DNA in mice: chromosomal association and placental transmission to the fetus. Mol. Gen. Genet. 259, 569–576 10.1007/s004380050850 9819049

[111] Benoit J., Leroy P., Vendrely R. and Vendrely C. (1960) Experiments on Pekin ducks treated with DNA from Khaki Campbell ducks. Trans. N. Y. Acad. Sci. 22, 494–503 10.1111/j.2164-0947.1960.tb00718.x 13798979

[112] Landman O.E. (1991) The inheritance of acquired characteristics. Annu. Rev. Genet. 25, 1–20 10.1146/annurev.ge.25.120191.000245 1812803

[113] Soucy S.M., Huang J. and Gogarten J.P. (2015) Horizontal gene transfer: building the web of life. Nat. Rev. Genet. 16, 472–482 10.1038/nrg3962 26184597

[114] Stroun M. and Anker P. (2005) Circulating DNA in higher organisms cancer detection brings back to life an ignored phenomenon. Cell. Mol. Biol. (Noisy-le-Grand) 51, 767–774 16359626

[115] Gahan P.B. and Stroun M. (2010) The virtosome–a novel cytosolic informative entity and intercellular messenger. Cell Biochem. Funct. 28, 529–538 10.1002/cbf.1690 20941743

[116] Stroun M., Lyautey J., Lederrey C., Olson-Sand A. and Anker P. (2001) About the possible origin and mechanism of circulating DNA apoptosis and active DNA release. Clin. Chim. Acta 313, 139–142 10.1016/S0009-8981(01)00665-9 11694251

[117] Csoka A.B. and Szyf M. (2009) Epigenetic side-effects of common pharmaceuticals: a potential new field in medicine and pharmacology. Med. Hypotheses 73, 770–780 10.1016/j.mehy.2008.10.039 19501473

[118] Kacevska M., Ivanov M. and Ingelman-Sundberg M. (2011) Perspectives on epigenetics and its relevance to adverse drug reactions. Clin. Pharmacol. Ther. 89, 902–907 10.1038/clpt.2011.21 21508940

[119] Kirk H., Cefalu W.T., Ribnicky D., Liu Z. and Eilertsen K.J. (2008) Botanicals as epigenetic modulators for mechanisms contributing to development of metabolic syndrome. Metabolism 57, S16–S23 10.1016/j.metabol.2008.03.006 18555849

[120] Knippen M.A. (2011) Microchimerism: sharing genes in illness and in health. ISRN Nurs 2011, 893819 21994897 10.5402/2011/893819 PMC3169192

[121] Shrivastava S., Naik R., Suryawanshi H. and Gupta N. (2019) Microchimerism: a new concept. J. Oral Maxillofac. Pathol. 23, 311 31516258 10.4103/jomfp.JOMFP_85_17 PMC6714269

[122] Lee T.-H., Paglieroni T., Utter G.H., Chafets D., Gosselin R.C., Reed W. et al. (2005) High-level long-term white blood cell microchimerism after transfusion of leukoreduced blood components to patients resuscitated after severe traumatic injury. Transfusion 45, 1280–1290 10.1111/j.1537-2995.2005.00201.x 16078913

[123] Reed W., Lee T.H., Norris P.J., Utter G.H. and Busch M.P. (2007) Transfusion-associated microchimerism: a new complication of blood transfusions in severely injured patients. Semin. Hematol. 44, 24–31 10.1053/j.seminhematol.2006.09.012 17198844

[124] Kunadian V., Zorkun C., Gibson W.J., Nethala N., Harrigan C., Palmer A.M. et al. (2009) Transfusion associated microchimerism: a heretofore little-recognized complication following transfusion. J. Thromb. Thrombolysis 27, 57–67 10.1007/s11239-008-0268-0 18766299

